# Cdk7 Is Required for Activity-Dependent Neuronal Gene Expression, Long-Lasting Synaptic Plasticity and Long-Term Memory

**DOI:** 10.3389/fnmol.2017.00365

**Published:** 2017-11-07

**Authors:** Guiqin He, Xiangyu Yang, Guo Wang, Junxia Qi, Rui Mao, Zhengping Wu, Zikai Zhou

**Affiliations:** ^1^Institute of Life Sciences, The Key Laboratory of Developmental Genes and Human Disease, Southeast University, Nanjing, China; ^2^School of Innovations, Sanjiang University, Nanjing, China; ^3^Co-innovation Center of Neuroregeneration, Nantong University, Nantong, China

**Keywords:** Cdk7, immediate early gene, synpatic plasticity, long-term memory, THZ1

## Abstract

In the brain, *de novo* gene expression driven by learning-associated neuronal activities is critical for the formation of long-term memories. However, the signaling machinery mediating neuronal activity-induced gene expression, especially the rapid transcription of immediate-early genes (IEGs) remains unclear. Cyclin-dependent kinases (Cdks) are a family of serine/threonine kinases that have been firmly established as key regulators of transcription processes underling coordinated cell cycle entry and sequential progression in nearly all types of proliferative cells. Cdk7 is a subunit of transcriptional initiation factor II-H (TFIIH) and the only known Cdk-activating kinase (CAK) in metazoans. Recent studies using a novel Cdk7 specific covalent inhibitor, THZ1, revealed important roles of Cdk7 in transcription regulation in cancer cells. However, whether Cdk7 plays a role in the regulation of transcription in neurons remains unknown. In this study, we present evidence demonstrating that, in post-mitotic neurons, Cdk7 activity is positively correlated with neuronal activities in cultured primary neurons, acute hippocampal slices and in the brain. Cdk7 inhibition by THZ1 significantly suppressed mRNA levels of IEGs, selectively impaired long-lasting synaptic plasticity induced by 4 trains of high frequency stimulation (HFS) and prevented the formation of long-term memories.

## Introduction

Neuronal gene expression is subject to highly dynamic regulations driven by neuronal activities. In particular, the rapid *de novo* expression of immediate-early genes (IEGs) such as *Npas4, Egr1, c-Fos, Nr4a1* and *Arc*, can be selectively triggered in subsets of neurons participating learning and memory formation following behavioral tasks (Morgan and Curran, 1989; Kubik et al., 2007; Lin et al., 2008; Korb and Finkbeiner, 2011; Ramamoorthi et al., 2011; Minatohara et al., 2016).

It is now widely accepted that action potentials generated during learning process are able to initiate gene transcription in a short period of time by inducing influxes of calcium into the neuron (Hardingham et al., 1997; Adams and Dudek, 2005). RNA polymerase II (RNAPII) stalling within the promoter-proximal region of genes refers to a state that after initiating RNA synthesis, RNAPII stalls after transcribing 20–50 nucleotides (Gilmour and Lis, 1986; Rougvie and Lis, 1988). This phenomenon has been proposed to be widespread and occurs across the genome (Muse et al., 2007). It is thought that RNAPII stalling is mediated by negative elongation factor (NELF) and is critical for near-instantaneous induction of IEGs in response to neuronal activity in mammalian neurons (Saha et al., 2011), and such a mechanism was shown to be critical to poise genes for rapid and synchronous induction (Zeitlinger et al., 2007; Boettiger and Levine, 2009; Saha et al., 2011), for the precise timing of IEG responses with their respective nuclear and synaptic functions.

Cyclin-dependent kinases (Cdks) are serine/threonine kinases that are critically involved in the regulation of transitions between different cell cycle phases. They interact with cyclins, the regulatory subunits providing domains essential for enzymatic activity and substrate specificity of Cdks (Guo and Price, 2013; Hydbring et al., 2016). Conceptually similar to the single Cdk in yeast, mammalian cells utilize a complex combination of Cdks and cyclins, such as Cdk4/cyclinD and Cdk2/cyclinE in the G1/S transition, and Cdk2/cyclinA and Cdk1/cyclinB in the G2/M transition, to regulate the cell division cycle (Larochelle et al., 2012).

It has now been firmly established that Cdk-mediated regulation of gene transcription underlies coordinated cell cycle entry and sequential progression in nearly all tested types of proliferative cells. In addition to such canonical role as key regulators of the cell cycle, Cdks and cyclins play non-canonical roles in DNA damage repair, proteolytic degradation, epigenetic regulation and metabolism (Guo and Price, 2013; Hydbring et al., 2016). Based on their functional correlations, mammalian Cdks can be categorized into cell-cycle-related (Cdk1, Cdk4, Cdk5) and transcriptional (Cdk7, Cdk8, Cdk9, Cdk11 and Cdk20) subfamilies. Cdk7 is a subunit of transcriptional initiation factor II-H (TFIIH), thus plays a major role in transcription initiation by phosphorylating the Ser 5 residue of the RNAPII C-terminal domain (CTD; Glover-Cutter et al., 2009; Sansó and Fisher, 2013). It also forms the Cdk-activating kinase (CAK) complex by binding with cyclin H and Mat 1, then phosphorylates the T-loops of all Cdks tested, thus activates Cdks (Sansó and Fisher, 2013; Harlen and Churchman, 2017).

However, despite extensive studies on the roles of Cdks in cells undergone frequent cell cycles, such as cancer cells and stem cells, the role of Cdk7 in the regulation of transcription remains unknown in post-mitotic neurons. In this study, by taking advantage of a recently developed novel Cdk7 specific covalent inhibitor THZ1, which negatively impacts gene expression and proliferation in cancer cells and animal models (Chipumuro et al., 2014; Christensen et al., 2014; Kwiatkowski et al., 2014; Nilson et al., 2015), we provide evidence demonstrating that Cdk7 activity is critical for neuronal activity-induced transcription of IEGs, long-lasting synaptic plasticity and the formation of long-term memories.

## Materials and Methods

### Animals

C57BL/6 mice were housed under a standard 12-h light/12-h dark cycle condition at the Experimental Animal Center at Southeast University, China. All animal experiment procedures were approved by Southeast University Animal Care and Use Committee. All procedures strictly followed the animal protocol (code: 20120926001) approved by the Southeast University Animal Care and Use Committee.

### Neuronal Culture and Drug Treatments

Cortical cultures were prepared from postnatal day 1 (P1) pups as previously described (Liu et al., 2016; Xia et al., 2016). Briefly, pups were sacrificed and cortical regions were dissected in ice-cold PBS. Tissues were digested by papain at 37°C for 15 min, dissociated by trituration and plated onto poly-D-lysine (50 μg/ml) coated glass coverslips (60,000 cells/ml). The cultures were maintained by replacing half of the medium with fresh medium every 4 days. The maintenance medium contained Neurobasal A, 0.5 mM GlutaMax and B27. At 14–15 DIV, cultured neurons were treated with THZ1, tetrodotoxin (TTX; 2 μM) or Bicucullin (25 μM) as indicated in each experiment. For protein lysate preparation, neurons were quickly washed with in PBS and lysed with ice-cold RIPA lysis buffer (Beyotime) with protease inhibitor cocktail and phosphatase inhibitor (Roche) and lysed for 20 min on ice.

### Western Blot

Proteins were extracted from mice cortex using lysis buffer containing 20 mM Tris (pH 7.5), 150 mM NaCl, 1 mM EDTA, 1 mM EGTA, 1% Triton X-100, 2.5 mM sodium pyrophosphate, 1 mM β-glycerophosphate, 1 mM Na_3_VO_4_, 20 mM NaF, and 1% protease inhibitor cocktail and phosphatase inhibitor (Roche). Debris was removed by centrifugation at 14,000 *g* (4°C) for 10 min. Protein samples were separated on SDS-PAGE and electrotransfered to a PVDF membrane. Membranes were blocked with 5% dry milk for 1 h and incubated with primary antibodies in TBST overnight at 4°C. After washing and incubation with secondary antibodies for 1 h, membranes were visualized with the chemiluminescence substrate (Thermo Fisher Scientific) and detected by ImageQuant™ LAS 4000 (GE Healtcare Life Sience). Primary antibodies were used as follows: anti-CDK7 (TA323115S, rabbit, 1:1000; Origene), anti-RNAPII CTD repeat YSPTSPS (phospho S2; ab193468, rabbit, 1:5000; Abcam), anti-RNAPII CTD repeat YSPTSPS (phospho S5; ab193467, rabbit, 1:2000; Abcam), anti-RNAPII CTD repeat YSPTSPS (phospho S7; ab126537, rabbit, 1:2000; Abcam), anti-tubulin (T9026, mouse, 1:5000; Sigma-Aldrich). Secondary antibodies were used as follows: goat anti-rabbit (A00098, 1:2000; Genscript), goat anti-mouse (A00160, 1:2000; Genscript). Preparation of CA1 tissue punch lysates for SDS-PAGE: hippocampi slices were isolated and were allowed for recovery at 28°C for at least 2 h in a submersion chamber perfused with 95% O_2_/5% CO_2_ saturated artificial cerebrospinal fluid (ACSF). Slices of hippocampi were pre-treated with THZ1 for 4 h before stimulations. After early-phase long-term potentiation (E-LTP) or late-phase LTP (L-LTP) stimulation, CA1 areas were removed for preparing protein samples and SDS-PAGE assay.

### Real Time PCR

Total RNA was isolated from mice cortical sections or cultured cortical neurons using Trizol reagent (Sangon Biotech) according to the manufacturer’s instructions. Total RNA (1 μg) of each sample was reverse transcribed into cDNA by using the Hiscript II 1st Strand cDNA Synthesis Kit (Vazyme Biotech). Then the cDNA were amplified by using a SYBR Green Master Mix Kit (Vazyme Biotech) in the CFX96 real-time PCR system (Bio-Rad). The amplify cycle was used as follows: 50°C for 2 min, 95°C for 10 min, and 40 cycles of 95°C for 15 s, 59°C for 1 min. The target genes expression levels were analyzed using the 2^−ΔΔCt^ method in which the target RNA was adjusted to that of *Gapdh*. Equations were used as follows: ΔCt = Ct Target − Ct *Gapdh*, ΔΔCt = ΔCt Treat − ΔCt Ctrl. mRNA levels of drug treatment groups were normalized to the mRNA levels of control groups that received equal duration of treatment with appropriate vehicles (e.g., DMSO as the control group to THZ1 as the experimental group).

The primers for target genes were used as follows: *Npas4*, forward: CTGCATCTACACTCGCAAGG, reverse: GCCACAATGTCTTCAAGCTCT; *cFos*, forward: ATGGGCTCTCCTGTCAACACAC, reverse: ATGGCTGTCACCGTGGGGATAAAG; *Arc*, forward: TACCGTTAGCCCCTATGCCATC, reverse: TGATATTGCTGAGCCTCAACTG; *Nr4a1*, forward: AGCCCAGGACCGCGTGACC, reverse: GGCAGCTGGTAGAGGAAGGTG; *Egr1*, forward: GGGAGCCGAGCGAACAA, reverse: CATTATTCAGAGCGATGTCAGAA; *c-Jun*, forward: TCCACGGCCAACATGCT, reverse: CCACTGTTAACGTGGTTCATGAC; *Homer1a*, forward: GAAGTCGCAGGAGAAGATG, reverse: TGATTGCTGAATTGAATGTGTACC; *Gapdh*, forward: AGGTCGGTGTGAACGGATTTG, reverse: TGTAGACCATGTAGTTGAGGTCA.

### Electrophysiology

Standard procedures for brain slices were described as previous study (Zhou et al., 2011). Briefly, the mouse brains were quickly dissected and transferred to ice-cold ACSF saturated with 95% O_2_/5% CO_2_. All slices were sagittaled to 360 μm. ACSF contained (in mM): 120.0 NaCl, 3.0 KCl, 1.0 NaH_2_PO_4_, 26.0 NaHCO_3_, 11.0 D-glucose, 1.2 MgSO_4_ and 2.0 CaCl_2_. The slices were recovered at 28°C for at least 2 h before a single slice was transferred to a submersion chamber perfused with 95% O_2_/5% CO_2_ saturated ACSF. Synaptic transmission was evoked by stimulation at 0.067 Hz (for field recordings) of Schaffer collaterals and recorded with glass pipettes (3–5 MΩ) filled with ACSF. For field recording: E-LTP was induced by two trains of high-frequency stimulation (HFS; 1 s, 100 Hz) stimulation, the interval between each train was 10 s. L-LTP was induced by four trains of HFS (1 s, 100 Hz) stimulation, the interval between each train was 5 min. All data acquisition and analysis were done using pCLAMP 10.2 (Axon Instruments). In all electrophysiological experiments, n represents the number of neurons or slices, and normally only one or two slices per animal were used.

### Behavioral Experiments

C57BL/6 male mice were housed up to five mice per cage in a 12-h light/12-h dark cycle. One injection cannula connected via a catheter to a Hamilton syringe (10 μl capacity) was aimed at the left lateral ventricle using the following coordinates: anteroposterior (AP) relative to Bregma, −0.5 mm; lateral (L) to midline, 1.0 mm; ventral (V) from the skull surface, −2.0 mm. Prior to tests, mice were recovered for a week and handled for 5 min a day for last 3 days. For the novel object recognition task, the mice were individually placed and accustomed to a square open field (50 cm × 50 cm × 50 cm) for 10 min for 4 days. THZ1-hydrochloride (20 μg per mouse, dissolved in DMSO at the concentration of 40 mM) and equal volume of DMSO (1 μl) were injected with 0.5 μl/min flow via the cannula controlled by a pump (KD Scientific, Legato 130) and the mice were awake. Three hours later, mice were exposed to two identical objects (object 1 and object 2) for 10 min, mice were returned to the field that now contained, the familiar object (object 2) and a novel object which was different in the size and color 2 h (for short term memory (STM)) or 24 h (for long term memory (LTM)) later. The mice were independent cohorts for STM and LTM. Time spent interacting with each object was manually analyzed in a blind manner. Discrimination index was calculated as % time with object 1/(time interaction with object 1 + time interaction with object 2) and novel object/(time interaction with novel object + time interaction with object 2). THZ1-hydrochloride (20 μg per mouse dissolved in DMSO at the concentration of 40 mM) and equal volume of DMSO (1 μl) were injected with 0.5 μl/min flow via the cannula controlled by a pump to allow the mice awake. Three hours later, each mouse was placed in a fear conditioning chamber and allowed to explore it for 3 min before the delivery of a 40 s tone (80 dB) which was immediately accompanied with a 2 s foot shock (0.7 mA) in the last 2 s of the tone, 40 s and 120 s later the second and third pairs of tone and foot shock stimulation were given. Mice were removed from the testing chambers 10 s after the third shock. During the cued test, the mouse was placed in a new chamber with different contextual cues for 2 h (for STM) or 24 h (for LTM) later. The mice were independent cohorts for STM and LTM. After a 3-min acclimation, the same tone used for training was presented without shocks, and freezing behavior was monitored for 4 min. Freezing behavior was analyzed for the percent of freezing time during the cued fear conditioning test. Percentage of freezing time was quantified using automated motion detection software Superfcs (Xinruan, China).

### Statistics

All the averaged data were stated as mean ± SEM. Statistical significance was assessed by one-way ANOVA with Bonferroni *post hoc* tests or by Student’s *t*-test, wherever applicable. All data analyzed were normally distributed. Results with *P* values of less than 0.05 were regarded as statistically significant, and * indicates *p* < 0.05, ** indicates *p* < 0.01, *** indicates *p* < 0.001 in the graphs.

## Results

### Expression of Cdk7 in the Mouse Cortex

**Figure 1:**
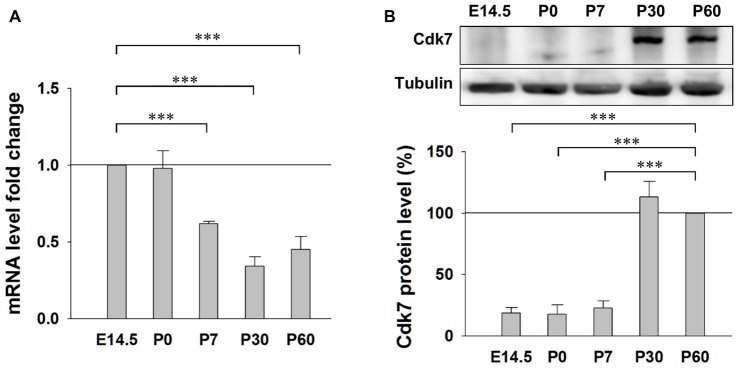
Expression of Cyclin-dependent kinase 7 (Cdk7) in the mouse cortex. **(A)** Quantitative RT-PCR results of Cdk7 mRNA levels in the mouse cortex at ages of E14.5, P0, P7, P30 and P60. mRNA level fold changes were normalized to that of E14.5. *n* = 4 mice per group. **(B)** Representative Western blot (WB) images and statistical results of Cdk7 protein levels in the cortex lysates prepared from mice at indicated ages. Data were normalized to that of P60. *n* = 3 mice per group. Data show means ± SEM. Statistical significance was assessed by one-way ANOVA with Bonferroni *post hoc* tests. ***p < 0.001.

First, we examined the expression pattern of Cdk7 in the brain at different ages in mice. Quantitative RT-PCR results showed similar levels of Cdk7 mRNA at embryonic day 14.5 (E14.5) and postnatal day 0 (P0), which gradually decreased to about 40% at the adult ages (P30 and P60; Figure 1A). However, in contrast to the gradual reduction at mRNA level, Cdk7 protein level significantly increased at adult ages in mice (Figure 1B), suggesting upregulated expression of Cdk7 at translational level when the mice mature.

### Irreversible Inhibition of Neuronal Cdk7 by THZ1

**Figure 2:**
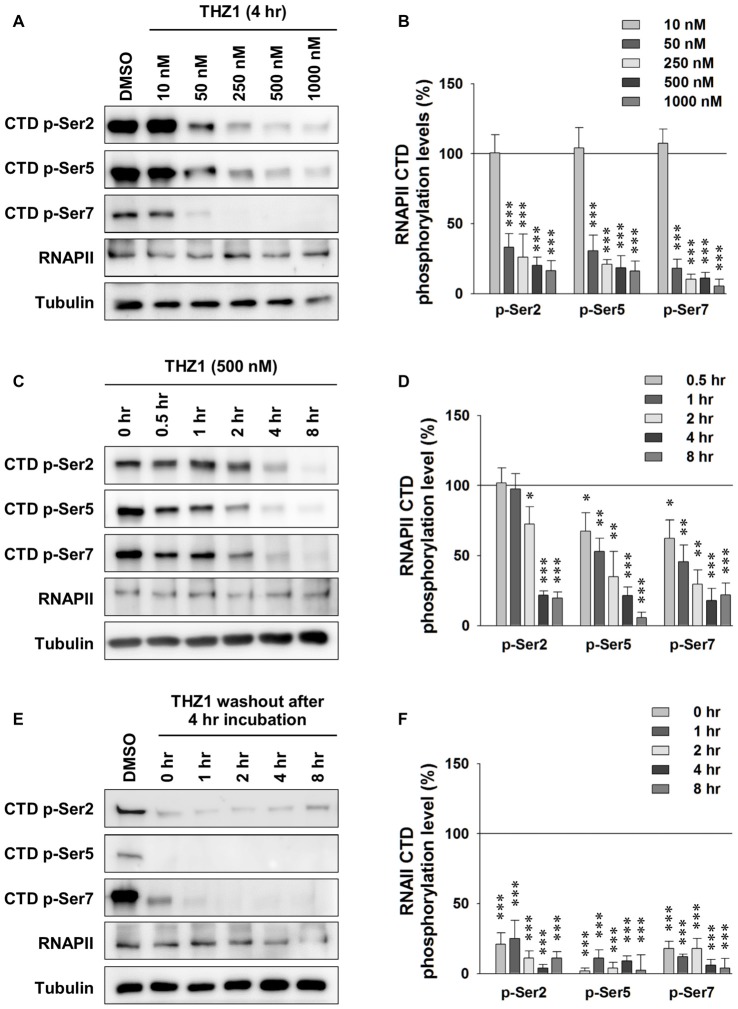
Irreversible inhibition of neuronal Cdk7 by THZ1. **(A)** Representative WB images and **(B)** statistical results of phosphorylation levels of residues Ser 2, Ser 5 and Ser 7 on RNA polymerase II (RNAPII)-C-terminal domain (CTD) after 4 h of THZ1 incubation at final concentrations of 10, 50, 250, 500 and 1000 nM in primary cultured neurons. *n* = 5 independent experiments per group. Phosphorylation levels were normalized to that of DMSO treatment group. **(C,D)** Representative WB images and statistical results showing the time course of THZ1 (500 nM)-induced reduction of phosphorylation levels of RNAPII-CTD from 0.5 to 8 h. *n* = 6 independent experiments per group. Phosphorylation levels were normalized to that of 0 h treatment group. **(E,F)** Representative WB images and statistical results showing the phosphorylation levels of RNAPII-CTD remained decreased for at least 8 h after 4 h of THZ1 (500 nM) incubation. *n* = 3 independent experiments per group. Data show means ± SEM. Phosphorylation levels were normalized to that of DMSO treatment group. Statistical significance in all panels was assessed by one-way ANOVA with Bonferroni *post hoc* tests. *p < 0.05, **p < 0.01, ***p < 0.001.

Cdk7 kinase activity has been implicated in the regulation of transcription by phosphorylating the CTD of RNAPII (Akhtar et al., 2009; Glover-Cutter et al., 2009). Therefore, we examined whether this process is conserved in neurons. We prepared primary mouse cortical neuronal cultures and examined the Cdk7 protein levels at days *in vitro* 1 (DIV 1), DIV 5, DIV 9 and DIV 15 by Western blot (WB; Supplementary Figure S1), we found gradual increase of Cdk7 amount as the cultured neurons mature. Then, we performed the following experiments at DIV 14–16. First, using WB, we probed phosphorylation levels of residues Ser 2, Ser 5 and Ser 7 on RNAPII CTD after 4-h incubation of THZ1 at final concentrations of 10, 50, 250, 500 and 1000 nM. The sample blots in Figure 2A showed that the phosphorylation status of residues Ser 2, Ser 5 and Ser 7 were most significantly reduced by THZ1 incubation at 500 and 1000 nM, and the inhibitory effects were comparable at these two concentrations (Figure 2B). Therefore, we chose 500 nM to delineate the time course of THZ1-induced inhibition of phosphorylation on the three Ser residues. As shown in Figure 2C, 500 nM of THZ1 significantly reduced the phosphorylation of RNAPII CTD 2 h after the onset of THZ1 incubation. This inhibition reached the maximal level at 4–8 h after the addition of THZ1 (Figure 2D). Thus, in primary cultured neurons, we have determined the time course and effective concentrations of THZ1 on neuronal Cdk7-mediated phosphorylation of residues Ser 2, Ser 5 and Ser 7 on RNAPII CTD.

Next, we performed washout experiments to test the irreversible inhibition of THZ1 to Cdk7 by covalent binding (Kwiatkowski et al., 2014) in neurons. The WB results show that the phosphorylation levels did not recover at least 8 h after the washout of THZ1 by conditioned media from sister cultures (Figures 2E,F). Together, these data demonstrate that: (1) Cdk7 is essential for the phosphorylation thus activation of RNAPII in neurons; (2) consistent with the results obtained in cancer cells and stem cells (Chipumuro et al., 2014; Christensen et al., 2014; Kwiatkowski et al., 2014; Nilson et al., 2015), in post-mitotic neurons, THZ1 effectively and irreversibly inhibits RNAPII CTD phosphorylation with similar potency via covalent binding to Cdk7.

### Cdk7 Activity Is Required to Maintain Gene Expression at Basal Level

At basal level, the synthesis of new mRNAs is important to maintain mRNA turnover for diverse physiological cellular events. Upon stimulation, synthesis of specific sets of new mRNAs, such as IEGs (*Npas4, Egr1, c-Fos, Nr4a1* and *Arc* etc.), are essential to consolidate newly acquired memory (Morgan and Curran, 1989; Kubik et al., 2007; Lin et al., 2008; Korb and Finkbeiner, 2011; Ramamoorthi et al., 2011). After the demonstration of Cdk7 expression in the brain (Figure 1), and the delineation of the temporal patterns of THZ1-mediated inhibition of Cdk7 and RNAPII in cultured neurons (Figure 2), we examined whether Cdk7 plays a role in neuronal gene expression at basal level and/or upon neuronal activity.

**Figure 3:**
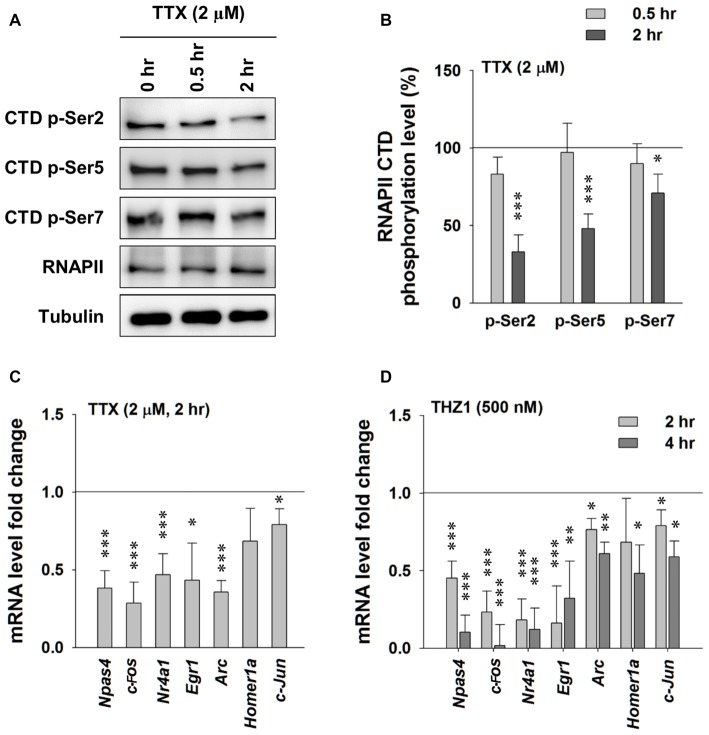
Tetrodotoxin (TTX)-induced neuronal activity deprivation reduces Cdk7 activity and reduces immediate-early gene (IEG) expression. **(A)** Representative WB images and **(B)** statistical results showing that TTX (2 μM, 2 h) treatment significantly reduced phosphorylation levels of residues Ser 2, Ser 5 and Ser 7 on RNAPII-CTD. *n* = 5 independent experiments per group. Phosphorylation levels were normalized to that of 0 h treatment group. **(C)** TTX (2 μM, 2 h) treatment significantly reduced mRNA levels of IEGs in primary cultured neurons. *n* = 5 independent experiments per group. mRNA levels were normalized to that of control group (i.e., the sister culture group treated with equal amount of the vehicle distilled H_2_O to that of TTX for 2 h). **(D)** THZ1 (500 nM) treatment for 2 or 4 h significantly reduced mRNA levels of IEGs. *n* = 5 independent experiments per group. mRNA levels were normalized to that of DMSO groups (i.e., the sister culture groups treated with equal amount of the vehicle DMSO to that of THZ1 for 2 h or 4 h, respectively). Data show means ± SEM. Statistical significance in **(B,D)** was assessed by one-way ANOVA with Bonferroni *post hoc* tests. Statistical significance in **(C)** was determined by Two-tailed Student’s *t*-test. *p < 0.05, **p < 0.01, ***p < 0.001.

First, we probed the alterations of phosphorylation levels of RNAPII CTD after activity-deprivation by blocking voltage-gated sodium channels with 2 μM TTX in cultured primary neurons. Figures 3A,B show that 2 h of TTX incubation induced moderate reduction in the phosphorylation levels of Ser 2, Ser 5 and Ser 7 on the RNAPII CTD, indicating reduced gene transcription. RT-PCR data show significantly decreased levels of *Npas4, Egr-1, c-Fos, Nr4a1, Arc* and *c-Jun* at 2 h after TTX addition (Figure 3C). Similarly, in the presence of THZ1, which induced drastic decrease of phosphorylation levels of Ser 2, Ser 5 and Ser 7 on the RNAPII CTD (Figures 2C,D), also drastically reduced IEG mRNA levels, especially *Npas4, c-Fos, Nr4a1* and *Egr1* (Figure 3D). Together, these data show that the decreased phosphorylation level of Cdk7 of RNAPII CTD, induced by either activity-deprivation or the inhibitor THZ1, significantly reduced the transcription of IEGs, suggesting an important role of Cdk7 activity in maintaining neuronal gene expression at basal level.

### Cdk7 Is Critical for Activity-Driven Expression of the Neuronal IEGs

**Figure 4:**
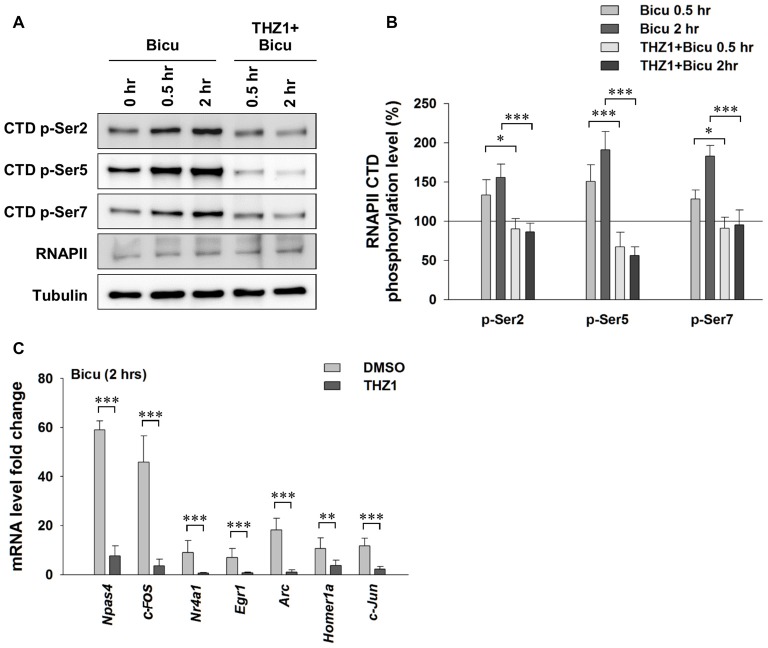
Neuronal activation-induced expression of IEGs requires Cdk7 activity. **(A)** Representative WB images and **(B)** statistical results showing that bicuculline (20 μM) treatment significantly increased phosphorylation levels of residues Ser 2, Ser 5 and Ser 7 on RNAPII-CTD in primary cultured neurons, and this increase can be prevented by pretreatment with Cdk7 inhibitor THZ1 (500 nM). *n* = 6 independent experiments per group. Phosphorylation levels were normalized to that of 0 h treatment group. **(C)** Bicuculline treatment drastically increased mRNA levels of IEGs, that were largely prevented by THZ1 (500 nM) pretreatment. *n* = 6 independent experiments per group. mRNA levels were normalized to that of control group (i.e., the sister culture group only treated with equal amount of the vehicle DMSO to that of THZ1 for 2 h). Data show means ± SEM. Statistical significance in **(B)** was assessed by one-way ANOVA with Bonferroni *post hoc* tests. Statistical significance in **(C)** was determined by Two-tailed Student’s *t*-test. *p < 0.05, **p < 0.01, ***p < 0.001.

Experience or pharmacological stimuli-induced neuronal activity can trigger Ca^2+^ influx, serving as the second messenger to initiate the IEG-dominated first wave of robust gene transcription, which leads to a global gene transcription cascade. Having examined the involvement of Cdk7 in gene expression at basal level, we next treated cultured primary neurons with 20 μM bicuculline, a specific GABA_A_ receptor antagonist (Figure 4A). After eliminating GABAergic inhibitory transmission, excitatory neurotransmission dominates in the neural network. This approach has been widely used to reliably induce neuronal activation in cell cultures. As shown in Figures 4A,B, bicuculline significantly increased the phosphorylation of Ser 2, Ser 5 and Ser 7 on the RNAPII CTD, indicating enhanced Cdk7-regulated gene expression. Next, we performed RT-PCR to test IEG mRNA levels (Figure 4C). The results show drastically unregulated IEG mRNA levels, up to ~60 fold. Importantly, bicuculline failed to increase RNAPII CTD phosphorylation in cultured primary neurons that had been pre-incubated with THZ1 (500 nM) for 4 h (Figures 4A,B). Bicuculine-induced drastic upregulation of IEG mRNA levels were largely prevented by THZ1 pre-incubation (Figure 4C). Thus, we conclude that Cdk7 activity is positively correlated to neuronal activities, and is critical for activity-induced neuronal gene expression.

### Cdk7 Activity Is Essential to Long-Lasting Synaptic Plasticity

Synaptic plasticity, an experimental phenomena observed at electrophysiological level, is widely believed to be the cellular and molecular mechanism underlying learning and memory. Based on different patterns of plasticity expression and key signaling molecules, it can be categorized to diverse forms, such as NMDAR-dependent LTP and long-term depression (LTD). So far, we have demonstrated the critical role of Cdk7 in activity-driven gene expression of IEGs. Next, to address the question whether Cdk7-regulated transcription underlies the forms of synaptic plasticity that require gene expression, we performed electrophysiological recordings in the CA1 region of the hippocampal Schaffer collateral pathway. In this pathway, two distinct phases of LTP, namely E-LTP and L-LTP, exist and are thought to be important for STM and LTM, respectively (Nguyen et al., 1994; Nayak et al., 1998; Bannerman et al., 2014). Importantly, transcription has been demonstrated to be critical for L-LTP, but not E-LTP (Nguyen et al., 1994; Nayak et al., 1998; Bannerman et al., 2014).

**Figure 5:**
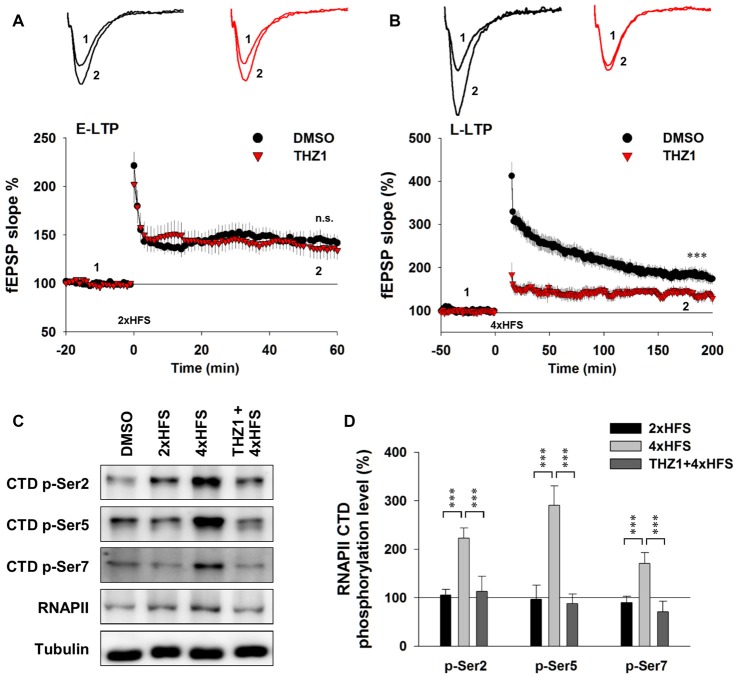
THZ1 selectively inhibited transcription-dependent late-phase LTP (L-LTP). **(A)** Early-phase LTP (E-LTP) induced by two trains of high-frequency stimulation (HFS) was not altered by pre-incubation of Cdk7 inhibitor THZ1 (500 nM, 4 h). *n* = 6 slices per group. **(B)** L-LTP induced by four trains of HFS 100 Hz lasting 1 s each delivered at 5-min intertrain intervals was significantly reduced by THZ1 pre-incubation. *n* = 7 slices per group. **(C)** Representative WB images and **(D)** statistical results showing that 4xHFS stimulation significantly increased the phosphorylation levels of residues Ser 2, Ser 5 and Ser 7 on RNAPII-CTD in acute hippocampal slices, and this increase was prevented by pretreatment with Cdk7 inhibitor THZ1 (500 nM). *n* = 5 independent experiments per group. Data show means ± SEM. Phosphorylation levels were normalized to that of DMSO group. Statistical significance was determined by Two-tailed Student’s *t*-test. ***p < 0.001.

To examine the involvement of Cdk7 in E-LTP and L-LTP, we pre-incubated acute hippocampal slices in the perfusate containing 500 nM THZ1 or equivalent volume of DMSO for 2 h before fEPSP recordings. Figure 5A shows that two trains of HFS (100 Hz lasting 1 s) delivered to the Schaffer collateral pathway, a protocol typically used to induce hippocampal E-LTP, successfully induced potentiation of fEPSP with similar magnitude in both DMSO and THZ1 groups at the last 10 min of recordings. However, four trains of HFS with 5 min inter-train intervals (4xHFS), a protocol typically used to induce L-LTP, induced robust potentiation of the evoked fEPSP slope lasting at least 3 h after the stimulation in the DMSO group, but the magnitude of potentiation was significantly reduced throughout the recordings after tetanus stimulation in slices pre-incubated in 500 nM THZ1 (Figure 5B). This is noteworthy that the first 1–2 h of L-LTP induced by 4xHFS does not require transcription (Nguyen et al., 1994). The above results suggest that THZ1-mediated Cdk7 inhibition may affect signaling pathways that play a role in the induction of L-LTP, other than transcription.

To confirm that the observed reduction of LTP induced by 4xHFS was caused by inhibition of Cdk7 by THZ1, we performed WB on tissue punch lysates of the hippocampal CA1 region. As Figure 5C shows, while two trains of HFS only moderately increased phosphorylation level of Ser 2 on RNAPII CTD, four trains of HFS drastically increased phosphorylation levels of residues Ser 2, Ser 5 and Ser 7. Moreover, in slices pre-incubated in 500 nM THZ1, the increased phosphorylation was largely prevented (Figures 5C,D).

### Cdk7 Activity Is Necessary for the Formation of Long-Term Memory

**Figure 6:**
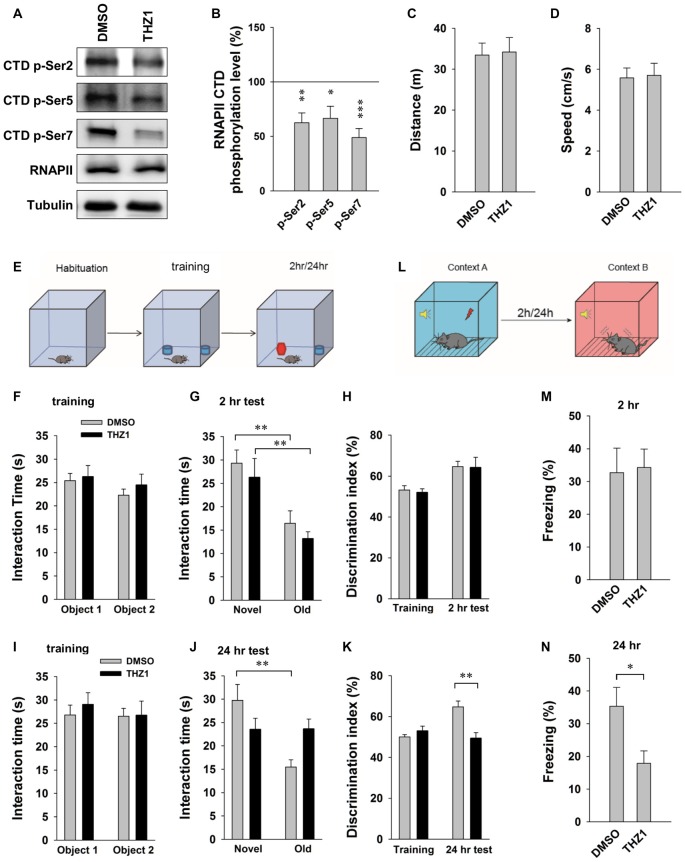
THZ1 treatment affects the formation of long-term memory (LTM). **(A)** Representative WB images and **(B)** statistical results showing that injection of 20 μg of THZ1 per brain significantly inhibited phosphorylation levels of residues Ser 2, Ser 5 and Ser 7 on RNAPII-CTD. Brain lysates were prepared 6 h after the injection, the time point corresponding to the training session. *n* = 6 independent experiments per group. Phosphorylation levels were normalized to that of DMSO group. **(C)** THZ1 injection did not affect traveled distance and **(D)** travel speed of mice. **(E)** Paradigm of novel object-recognition task. **(F)** Mice received THZ1 injection did not show difference in interaction time between object 1 and object 2. **(G)** In short-term novel object-recognition tests performed 2 h after training, both DMSO and THZ1 groups showed significant preference to the novel object. **(H)** Both groups showed similar discrimination index in training and 2 h test sessions. **(I)** DMSO and THZ1 groups showed similar interaction time in training session, but only DMSO group showed preference to novel object in long-term novel object recognition tests performed 24 h after training **(J)**, resulting in higher discrimination index **(K)** when compared to the THZ1 group. **(L)** Paradigm of fear conditioning test. **(M)** Mice received THZ1 showed similar amount of freezing response when compared to DMSO group in short-term test (2 h post training), but showed significantly reduced freezing time in **(N)** LTM test (24 h post training). *n* = 10 mice per group in all behavioral tests. Data show means ± SEM. Statistical significance was determined by Two-tailed Student’s *t*-test. *p < 0.05, **p < 0.01, ***p < 0.001.

Behavioral tasks are known to trigger robust and near-instantaneous IEG expression in specific neuronal ensembles that presumably encode specific memories. Results of the above experiments have established an important role of Cdk7 in controlling the expression of IEGs driven by neuronal activities, and that THZ1 can effectively inhibit such gene expression and prevent L-LTP by inhibiting Cdk7 activity. Next, we turned to behavioral analysis to determine whether THZ1-mediated Cdk7 inhibition affects the forms of memory that requires gene expression. Before performing behavioral tests, we examined the inhibitory effects of Cdk7 by THZ1 in the brain of live mice. Six hours after injection of 20 μg of THZ1 or equal volume of DMSO through implanted cannula connected via a catheter, we prepared whole brain lysates and probed the phosphorylation levels of RNAPII CTD. As shown in Figures 6A,B, THZ1 injection significantly reduced Ser 2, Ser 5 and Ser 7 phosphorylation on RNAPII CTD at this time point corresponding to the training session.

For behavioral tests, after habituation for 4 days, 10 min each day, different cohorts of mice were injected with THZ1 or DMSO through implanted cannula at 3 h prior to training. When compared to DMSO, THZ1 did not affect traveled distance and speed (Figures 6C,D), suggesting that THZ1 does not alter general mobility of the mice. We next carried out a novel object-recognition task, in which mice were briefly exposed to two identical objects during the training session, followed by object-recognition tests 2 h (short-term) or 24 h (long-term) later, respectively (Figure 6E). In the test, trained mice were exposed to one novel and one familiar object. The rationale is that if mice remember the familiar object, they should spend less time with it and more time with the novel object. The results in Figure 6F show that DMSO and THZ1 groups spent similar time to explore the two identical objects. When mice were tested 2 h after the initial exposure, the group received THZ1 injection exhibited preference to the novel object similar to control group received DMSO injection, as measured by interaction time (Figure 6G) and discrimination index (Figure 6H). Similarly, in independent cohorts of mice, DMSO and THZ1 group did not show difference at the training session (Figure 6I), but only the DMSO group exhibited significantly more interaction time on the novel object tested 24 h later (Figure 6J), resulting in higher discrimination index when compared to THZ1 group (Figure 6K). These data suggest that THZ1 does not disrupt learning process or STM as evidenced by the tests 2 h after exposure, but specifically affected LTM, which requires gene transcription.

To further assess THZ1-induced impairments in cognitive performance, we used fear conditioning paradigm (Figure 6L) to examine long-term fear memory in mice. In the tests performed 2 h (Figure 6M) after the training, a paradigm generally considered as STM, mice received THZ1 showed similar amount of freezing response when compared to DMSO group (Figure 6M), suggesting that these two groups of mice learned equally well during training session and were comparable in STM. However, when cued fear conditioning tests were performed 24 h after training, a paradigm generally considered as LTM that requires transcription, mice received THZ1 froze significantly less than mice received DMSO (Figure 6N), indicating selective impairment in LTM. These behavioral data provide *in vivo* evidence supporting an important role of Cdk7 in the regulation of transcription-dependent neuronal functions.

## Discussion

Our results demonstrate that Cdk7 is differentially expressed in the mammalian brain at different development stages. The phosphorylation levels of Ser 2, Ser 5 and Ser 7 on RNAPII CTD positively correlate with neuronal activities, which can be drastically reduced by THZ1, a recently developed covalent inhibitor specific to Cdk7. It largely and irreversibly inhibited Cdk7 activity in cultured primary neurons, in a temporal pattern and at a concentration similar to that in stems cells and cancer cells (Kwiatkowski et al., 2014). As a result, THZ1 significantly inhibited activity-driven IEG transcription in cultured primary neurons, long-lasting synaptic plasticity in acute hippocampal slices and LTM in live mice. Together, we demonstrate that Cdk7 activity is critical for activity-driven transcription and neuronal functions. To our knowledge, up to present, the only study regarding Cdk7 in the brain is an earlier report showing increased CDK7 immunoreactivity in susceptible hippocampal neurons of Alzheimer’s disease patients (Zhu et al., 2000). Therefore, this study provides the first evidence that demonstrates a conserved role of Cdk7-regulated gene expression in post-mitotic neurons.

External stimuli, such as behavioral tasks, can initiate IEG-dominated first wave of gene transcription in subsets of neurons responsive to the stimuli. Since many IEGs encode transcription factors, they may directly regulate transcription of the second wave of effector genes. Newly synthesized proteins are required for synaptic plasticity, eventually memory formation. The highly synchronized IEG transcription is thought to be mediated by RNAPII stalling within the promoter-proximal region of genes (Zeitlinger et al., 2007; Boettiger and Levine, 2009; Saha et al., 2011). Saha et al., demonstrated that, in neurons, RNAPII stalling is mediated by NELF and is critical for near-instantaneous induction of IEGs in response to neuronal activity (Saha et al., 2011). However, THZ1-induced inhibition of IEG transcription in this study may not be solely attributable to defects in RNAPII stalling. Using an *in vitro* transcription system with Hela nuclear extract (Nilson et al., 2015) demonstrated that THZ1 not only causes defects in RNAPII CTD phosphorylation and promoter proximal stalling, but also in co-transcriptional capping and productive elongation, meanwhile with minimal effects on transcription initiation, thus revealed unexpected function of Cdk7 in RNAPII stalling and mRNA capping (Coin and Egly, 2015). It is interesting to examine whether the above regulatory machinery is conserved in post-mitotic neurons.

Transcription of IEGs in neurons is known to be highly sensitive to neuronal activity and is critical for LTM formation. Long-lasting forms of synaptic plasticity such as LTP are thought to be fundamental cellular mechanisms underlying learning and memory. Induction of LTP has been shown to occur concomitantly with learning in the hippocampi of live mice, and learning-induced synaptic potentiation can preclude subsequent electrically-induced LTP (Whitlock et al., 2006), indicating an important role of synaptic plasticity in memory formation. Importantly, transcription has been demonstrated to be critical for L-LTP, but not E-LTP (Nguyen et al., 1994; Nayak et al., 1998; Bannerman et al., 2014). Our electrophysiological recordings show that THZ1 pre-incubation did not alter E-LTP, but largely impaired fEPSP potentiation at both the induction and maintenance stages of L-LTP. In consistency, we found that THZ1 administration did not affect short-term object memory or cued fear memory, but impaired long-term object memory and cued fear memory. Thus, this study is the first report demonstrating a pivotal role of Cdk7 in the expression of long-lasting form of synaptic plasticity and LTM. However, please note that because THZ1 requires several hours to inhibit RNAPII activity (Figure 2C) and the binding of THZ1 to Cdk7 is covalent and irreversible, therefore, we could not selectively inhibit Cdk7 at different stages of L-LTP by applying timed perfusion of THZ1. The development of new Cdk7 inhibitor may be necessary to address this question. Moreover, the observation that Cdk7 inhibition did not affect E-LTP induced by 2xHFS, but significantly suppressed fEPSP potentiation at both the induction and maintenance stages of long-lasting LTP induced by 4xHFS, suggests that, other than transcriptional regulation, Cdk7 may regulate certain signaling molecules that are required for the first 1–2 h of LTP expression induced by 4xHFS.

Our results show that Cdk7 activity is positively correlated with neuronal activities in cultured primary neurons, acute hippocampal slices and in the brain. However the signaling pathway linking neuronal activities to Cdk7 activity remain elusive. Given the fact that experience or pharmacological stimuli-induced neuronal activity can trigger Ca^2+^ influx, serving as the second messenger to initiate the IEG-dominated first wave of robust gene transcription, we believe that Ca^2+^ influx plays an indispensable role in this pathway. In addition, mammalian cells are known to utilize a complex combination of Cdks and cyclins to regulate cell cycle (Larochelle et al., 2012). Cdk7 can form the CAK complex by binding with cyclinH and Mat1, then phosphorylates the T-loops of all Cdks tested, thus activates Cdks (Sansó and Fisher, 2013; Harlen and Churchman, 2017). It is interesting to examine whether the formation of Cdk7/cyclinH/Mat1 complex is subject to the regulation of Ca^2+^ influx driven by neuronal activities in future studies.

Brd4, a member of bromo domain and extra-terminal domain (BET) protein family, binds acetylated histones and regulates general transcription in many cell types. Similar to the effects of THZ1-mediated Cdk7 inhibition examined in this study, Brd4 inhibitor Jq1 significantly prevented IEG transcription, synaptic modifications and LTM in mice (Korb et al., 2015). Moreover, it is very interesting that its inhibition by Jq1 has been demonstrated to decrease seizure susceptibility (Korb et al., 2015), potentially by decreasing levels of the GluA1 subunit of AMPARs, the major mediators of fast excitatory synaptic transmission. Indeed, most current epilepsy treatments directly target existing synaptic proteins and receptors, but transcription inhibitors can reduce overall synaptic protein level and may provide a more robust method of dampening the heightened synaptic activity that leads to seizures (Korb et al., 2015). Hypothetically, THZ1 may exert therapeutic effects comparable to Jq1, in terms of decreasing seizure susceptibility. It is interesting to address this possibility in future studies. Moreover, the covalent feature of THZ1 provides unique advantage over reversible inhibitors in that repeated drug administration is unnecessary.

CDK7 inhibitor THZ1 has been proposed as potential treatment for several types of cancer (Chipumuro et al., 2014; Christensen et al., 2014; Kwiatkowski et al., 2014). Although we and others did not observe obvious deficits in the health or general mobility of mice, our results show that Cdk7 is highly expressed the brain, and THZ1 treatment causes memory deficits in mice. Therefore caution should be taken in potential clinical trials to avoid risk of neurological side effects.

## Author Contributions

GH: performed the experiments, analysis and interpretation of data, revising the article; XY: performed the experiments, analysis and interpretation of data; GW, JQ, RM and ZW: performed the experiments, analysis of data; ZZ: conception and design, interpretation of data, designed the study, wrote the article.

## Conflict of Interest Statement

The authors declare that the research was conducted in the absence of any commercial or financial relationships that could be construed as a potential conflict of interest.

## Supplementary Material

Figure S1 Expression of Cyclin-dependent kinase 7 (Cdk7) in cultured primary neurons. **(A)** Representative Western blot images and statistical results of Cdk7 protein levels in the cultured primary neurons. Lysates were prepared from neurons at DIV1, DIV5, DIV9 and DIV15. Protein levels were normalized to that of DIV1. *n* = 5 independent experiments per group. Data show means ± SEM. Statistical significance was assessed by one-way ANOVA with Bonferroni *post hoc* tests. **p* < 0.05.

## References

[B1] Adams J. P. Dudek S. M. (2005). Late-phase long-term potentiation: getting to the nucleus. Nat. Rev. Neurosci. 6, 737–743. 10.1038/nrn1749 16136174

[B2] Akhtar M. S. Heidemann M. Tietjen J. R. Zhang D. W. Chapman R. D. Eick D.. (2009). TFIIH kinase places bivalent marks on the carboxy-terminal domain of RNA polymerase II. Mol. Cell 34, 387–393. 10.1016/j.molcel.2009.04.016 19450536 PMC2757088

[B3] Bannerman D. M. Sprengel R. Sanderson D. J. McHugh S. B. Rawlins J. N. Monyer H.. (2014). Hippocampal synaptic plasticity, spatial memory and anxiety. Nat. Rev. Neurosci. 15, 181–192. 10.1038/nrn3677 24552786

[B4] Boettiger A. N. Levine M. (2009). Synchronous and stochastic patterns of gene activation in the *Drosophila* embryo. Science 325, 471–473. 10.1126/science.1173976 19628867 PMC4280267

[B5] Chipumuro E. Marco E. Christensen C. L. Kwiatkowski N. Zhang T. Hatheway C. M.. (2014). CDK7 inhibition suppresses super-enhancer-linked oncogenic transcription in MYCN-driven cancer. Cell 159, 1126–1139. 10.1016/j.cell.2014.10.024 25416950 PMC4243043

[B6] Christensen C. L. Kwiatkowski N. Abraham B. J. Carretero J. Al-Shahrour F. Zhang T.. (2014). Targeting transcriptional addictions in small cell lung cancer with a covalent CDK7 inhibitor. Cancer Cell 26, 909–922. 10.1016/j.ccell.2014.10.019 25490451 PMC4261156

[B7] Coin F. Egly J.-M. (2015). Revisiting the function of CDK7 in transcription by virtue of a recently described TFIIH kinase inhibitor. Mol. Cell 59, 513–514. 10.1016/j.molcel.2015.08.006 26295956

[B8] Gilmour D. S. Lis J. T. (1986). RNA polymerase II interacts with the promoter region of the noninduced hsp70 gene in *Drosophila melanogaster* cells. Mol. Cell. Biol. 6, 3984–3989. 10.1128/mcb.6.11.3984 3099167 PMC367162

[B9] Glover-Cutter K. Larochelle S. Erickson B. Zhang C. Shokat K. Fisher R. P.. (2009). TFIIH-associated Cdk7 kinase functions in phosphorylation of C-terminal domain Ser7 residues, promoter-proximal pausing, and termination by RNA polymerase II. Mol. Cell. Biol. 29, 5455–5464. 10.1128/MCB.00637-09 19667075 PMC2756882

[B10] Guo J. Price D. H. (2013). RNA polymerase II transcription elongation control. Chem. Rev. 113, 8583–8603. 10.1021/cr400105n 23919563 PMC4294624

[B11] Hardingham G. E. Chawla S. Johnson C. M. Bading H. (1997). Distinct functions of nuclear and cytoplasmic calcium in the control of gene expression. Nature 385, 260–265. 10.1038/385260a0 9000075

[B12] Harlen K. M. Churchman L. S. (2017). The code and beyond: transcription regulation by the RNA polymerase II carboxy-terminal domain. Nat. Rev. Mol. Cell Biol. 18, 263–273. 10.1038/nrm.2017.10 28248323

[B13] Hydbring P. Malumbres M. Sicinski P. (2016). Non-canonical functions of cell cycle cyclins and cyclin-dependent kinases. Nat. Rev. Mol. Cell Biol. 17, 280–292. 10.1038/nrm.2016.27 27033256 PMC4841706

[B14] Korb E. Finkbeiner S. (2011). Arc in synaptic plasticity: from gene to behavior. Trends Neurosci. 34, 591–598. 10.1016/j.tins.2011.08.007 21963089 PMC3207967

[B15] Korb E. Herre M. Zucker-Scharff I. Darnell R. B. Allis C. D. (2015). BET protein Brd4 activates transcription in neurons and BET inhibitor Jq1 blocks memory in mice. Nat. Neurosci. 18, 1464–1473. 10.1038/nn.4095 26301327 PMC4752120

[B16] Kubik S. Miyashita T. Guzowski J. F. (2007). Using immediate-early genes to map hippocampal subregional functions. Learn. Mem. 14, 758–770. 10.1101/lm.698107 18007019

[B17] Kwiatkowski N. Zhang T. Rahl P. B. Abraham B. J. Reddy J. Ficarro S. B.. (2014). Targeting transcription regulation in cancer with a covalent CDK7 inhibitor. Nature 511, 616–620. 10.1038/nature13393 25043025 PMC4244910

[B18] Larochelle S. Amat R. Glover-Cutter K. Sansó M. Zhang C. Allen J. J.. (2012). Cyclin-dependent kinase control of the initiation-to-elongation switch of RNA polymerase II. Nat. Struct. Mol. Biol. 19, 1108–1115. 10.1038/nsmb.2399 23064645 PMC3746743

[B19] Lin Y. Bloodgood B. L. Hauser J. L. Lapan A. D. Koon A. C. Kim T.-K.. (2008). Activity-dependent regulation of inhibitory synapse development by Npas4. Nature 455, 1198–1204. 10.1038/nature07319 18815592 PMC2637532

[B20] Liu A. Zhou Z. Dang R. Zhu Y. Qi J. He G.. (2016). Neuroligin 1 regulates spines and synaptic plasticity via LIMK1/cofilin-mediated actin reorganization. J. Cell Biol. 212, 449–463. 10.1083/jcb.201509023 26880202 PMC4754719

[B21] Minatohara K. Akiyoshi M. Okuno H. (2016). Role of immediate-early genes in synaptic plasticity and neuronal ensembles underlying the memory trace. Front. Mol. Neurosci. 8:78. 10.3389/fnmol.2015.00078 26778955 PMC4700275

[B22] Morgan J. I. Curran T. (1989). Stimulus-transcription coupling in neurons: role of cellular immediate-early genes. Trends Neurosci. 12, 459–462. 10.1016/0166-2236(89)90096-9 2479148

[B23] Muse G. W. Gilchrist D. A. Nechaev S. Shah R. Parker J. S. Grissom S. F.. (2007). RNA polymerase is poised for activation across the genome. Nat. Genet. 39, 1507–1511. 10.1038/ng.2007.21 17994021 PMC2365887

[B24] Nayak A. Zastrow D. J. Lickteig R. Zahniser N. R. Browning M. D. (1998). Maintenance of late-phase LTP is accompanied by PKA-dependent increase in AMPA receptor synthesis. Nature 394, 680–683. 10.1097/00001756-199804200-00041 9716131

[B25] Nguyen P. Abel T. Kandel E. (1994). Requirement of a critical period of transcription for induction of a late phase of LTP. Science 265, 1104–1107. 10.1126/science.8066450 8066450

[B26] Nilson K. A. Guo J. Turek M. E. Brogie J. E. Delaney E. Luse D. S.. (2015). THZ1 reveals roles for Cdk7 in Co-transcriptional capping and pausing. Mol. Cell 59, 576–587. 10.1016/j.molcel.2015.06.032 26257281 PMC4546572

[B27] Ramamoorthi K. Fropf R. Belfort G. M. Fitzmaurice H. L. McKinney R. M. Neve R. L.. (2011). Npas4 regulates a transcriptional program in CA3 required for contextual memory formation. Science 334, 1669–1675. 10.1126/science.1208049 22194569 PMC4038289

[B28] Rougvie A. E. Lis J. T. (1988). The RNA polymerase II molecule at the 5^′^ end of the uninduced hsp70 gene of D. melanogaster is transcriptionally engaged. Cell 54, 795–804. 10.1016/s0092-8674(88)91087-2 3136931

[B29] Saha R. N. Wissink E. M. Bailey E. R. Zhao M. Fargo D. C. Hwang J.-Y.. (2011). Rapid activity-induced transcription of Arc and other IEGs relies on poised RNA polymerase II. Nat. Neurosci. 14, 848–856. 10.1038/nn.2839 21623364 PMC3125443

[B30] Sansó M. Fisher R. P. (2013). Pause, play, repeat: CDKs push RNAP II’s buttons. Transcription 4, 146–152. 10.4161/trns.25146 23756342 PMC3977912

[B31] Whitlock J. R. Heynen A. J. Shuler M. G. Bear M. F. (2006). Learning induces long-term potentiation in the hippocampus. Science 313, 1093–1097. 10.1126/science.1128134 16931756

[B32] Xia S. Zhou Z. Leung C. Zhu Y. Pan X. Qi J.. (2016). p21-activated kinase 1 restricts tonic endocannabinoid signaling in the hippocampus. Elife 5:e14653. 10.7554/eLife.14653 27296803 PMC4907698

[B33] Zeitlinger J. Stark A. Kellis M. Hong J.-W. Nechaev S. Adelman K.. (2007). RNA polymerase stalling at developmental control genes in the *Drosophila melanogaster* embryo. Nat. Genet. 39, 1512–1516. 10.1038/ng.2007.26 17994019 PMC2824921

[B34] Zhou Z. Hu J. Passafaro M. Xie W. Jia Z. (2011). GluA2 (GluR2) regulates metabotropic glutamate receptor-dependent long-term depression through N-cadherin-dependent and cofilin-mediated actin reorganization. J. Neurosci. 31, 819–833. 10.1523/JNEUROSCI.3869-10.2011 21248105 PMC6632944

[B35] Zhu X. Rottkamp C. A. Raina A. K. Brewer G. J. Ghanbari H. A. Boux H.. (2000). Neuronal CDK7 in hippocampus is related to aging and Alzheimer disease. Neurobiol. Aging 21, 807–813. 10.1016/s0197-4580(00)00217-7 11124424

