# Supplementary figures and images for: Cdk7 Is Required for Activity-Dependent Neuronal Gene Expression, Long-Lasting Synaptic Plasticity and Long-Term Memory
Source: Front Mol Neurosci. 2017 Nov 7;10:365. doi: 10.3389/fnmol.2017.00365 (PMC5681959; doi:10.3389/fnmol.2017.00365)

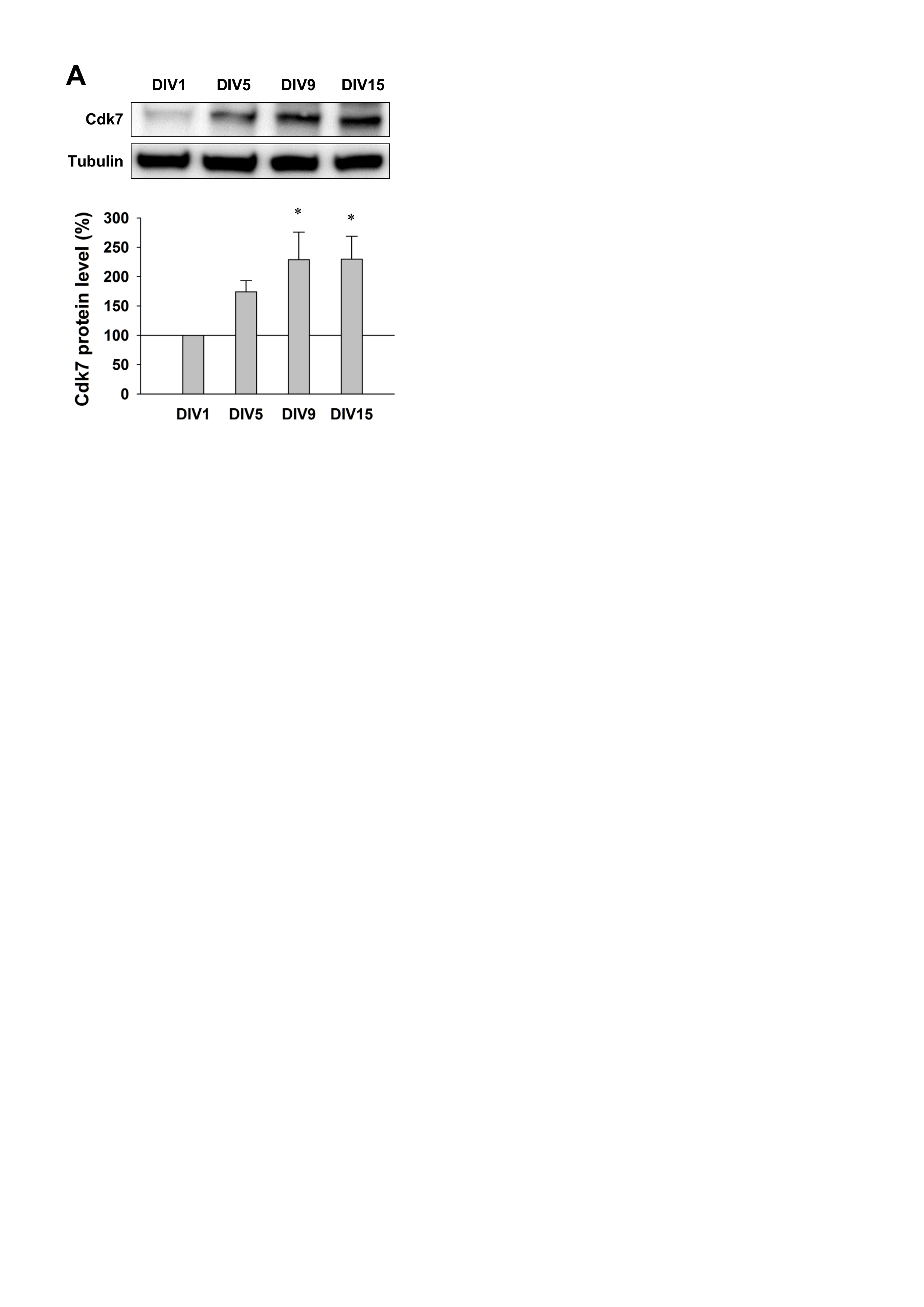

Supplement: Figure S1 — Expression of Cyclin-dependent kinase 7 (Cdk7) in cultured primary neurons. (A) Representative Western blot images and statistical results of Cdk7 protein levels in the cultured primary neurons. Lysates were prepared from neurons at DIV1, DIV5, DIV9 and DIV15. Protein levels were normalized to that of DIV1. n = 5 independent experiments per group. Data show means ± SEM. Statistical significance was assessed by one-way ANOVA with Bonferroni post hoc tests. *p < 0.05. [file Image_1.tif]
